# On Recording the Unipolar ECG Limb Leads via the Wilson's vs the Goldberger's Terminals: aVR, aVL, and aVF Revisited

**Published:** 2008-11-01

**Authors:** John E Madias

**Affiliations:** Mount Sinai School of Medicine of the New York University, and the Division of Cardiology, Elmhurst Hospital Center, New York, NY

**Keywords:** augmented unipolar limb leads, Wilson's central terminal, Goldberger's central terminal

The augmented unipolar limb leads aVR, aVL, and aVF, introduced by Goldberger in 1942, are an integral part of the 12-lead ECG [1,2].  Leads I, II, and III have 2 dedicated electrodes, but the other 9 leads have a single dedicated electrode, and another one constructed from the averaged inputs of multiple electrodes. This Viewpoint discusses whether an indifferent pole for the recording of unipolar limb leads is best provided by the Wilson's central terminal (WCT), or by inputs from 2 limb electrodes (Goldberger's central terminal) (GCT), as done currently, and whether the latter have any advantages over the former. The term "unipolar", popularized by Wilson, is a misnomer, since no leads can be truly "unipolar", all requiring positive and negative poles. Thus the term unipolar is used herein in the quasi-unipolar sense, as when first introduced by Wilson and Goldberger, who also realized that such leads were not truly unipolar. The popularity of the unipolar leads reflected the quest of recording the ECG from various vantage points of the body, considering the limitations of the 3 bipolar leads, introduced by Einthoven [3], which register the difference of 2 ECG curves recorded at the 2 poles of these leads, and no variation in potential at each of these poles [4].  In contrast the unipolar leads were thought to register such variation of absolute potential, something not really true. Initially the WCT was used to record the unipolar limb leads [5],  but the amplitude was low, and the inscribed ECGs, then, and for many decades later [6], were thick-lined (≥2 mm) (Figure 1).

Goldberger thought that the three 5,000 Ω resistances of the WCT were not necessary, and they could be substituted by 3 plain wires to form a terminal for recording of unipolar limb leads. He also reported that his limb leads ECGs were identical in morphology to the ones obtained via the WCT [1,2]. However his innovation consisted of disconnecting the electrode from the composition of his GCT of the unipolar lead he was recording. Thus when aVR is recorded the GCT consists of a connection of left arm and left leg; when aVL is recorded the GCT consists of a connection of right arm and left leg; when aVF is recorded the GCT consists of a connection of right arm and left arm [1,2]. Thus the GCT is variable, consisting of the mean of the potentials of the 2 (different for the 3 recordings) limb leads, in contrast to the WCT which is unvariable [1,2,5-8]. This modification of Goldberger leads to the augmentation of the recorded limb leads by 50%, as can be shown mathematically, and thus the aVR, aVL, and aVF came into being [1,2,7-9].

The WCT does not represent a zero potential [6], since it is ~0.3 mV [10]; also GCT carries not a zero potential since the high resistances at the skin-electrode interfaces are not equal [1]. Consequently aVR, aVL and aVF and V1-V6 leads are not unipolar, but bipolar leads, with the indifferent pole carrying a very low negative potential. The difference in the potential of the GCT and WCT is reflected in the difference of the voltages recorded by these 2 systems, the one recorded by the former being augmented.

Is this augmentation of any use, or we can go back [11] to acquiring the unipolar limb leads via the WCT? An ECG (Figure 2) was routinely recorded, and immediately repeated at the same calibration with the V1, V2, and V3 leads connected to the right arm, left arm and left leg, respectively. The V1, V2 and V3 of the 2nd ECG included now the leads VR, VL, and VF, recorded via the WCT; the morphology of the unipolar limb leads in both ECGs is the same, but their amplitude in the 2nd ECG, is attenuated by ~1/3.

Using the same approach, another ECG from a patient with marked peripheral edema, reveals that the morphology of the complexes in VR, VL, and VF (Figure 3) is well appreciated, and measurements can be easily carried out, even in the case of a low voltage ECG [12].

Although this Viewpoint deals with WCT and GCT, there are many other terminals either not implemented routinely, or used for other purposes than recording the standard ECG. In the former category one can consider as terminal an indifferent electrode attached to remote part of the body, where potentials from the heart's generator are weak; in the latter category the electrodes attached on the torso in the exercise ECG leads hook-up form a terminal and the varying positions on the thorax where electrodes are attached for the recording of 12-lead ambulatory ECGs also represent another terminal. For a detailed exposition on terminals, and leads, that may also have advantages from what have prevailed (e.g, Burger's concepts took into consideration that the human body is three-dimensional, irregularly shaped, bounded, and an inhomogeneous volume conductor) the reader should consult other sources [8].

The standard ECG consists of 3 different sets of leads: the bipolar leads I, II, and II, the unipolar V1-V6 leads [5,7-9,11] recorded via the stable WCT, and the unipolar aVR, aVL, and aVF leads recorded via the changing GCT [1,2]. The aberration is that the last are augmented, are acquired via a thrice changing terminal, and a different one, than the WCT. A particular heart's zero potential, also by itself constantly changing in 3D space, must be one, and thus having both the WCT and GCT used does not appear theoretically appealing. For uniformity's sake it would be preferable to record V1-V6 and the 3 unipolar limb leads via the WCT. In this fashion the ECG would be similar in derivation to body surface maps, employing multiple thoracic unipolar leads, recorded via the WCT [8,9].  Accordingly a 9-unipolar lead ECG consisting of the V1-V6 leads, and the 3 unipolar limb leads, obtained via the WCT would suffice. The bipolar leads I, II, and III do not belong in such an idealized schema, but their historic importance, and their association with so many useful applications, supports their retention; nevertheless these leads can be supplanted by the 3 unipolar limb leads with impunity. In fact there is a mathematical relation between the bipolar and unipolar limb leads [8,9], and this is exploited by the modern electrocardiographs, which record I and II and calculate the other 4 limb leads on line [8].

Are there any conceivable drawbacks from such a substitution? In reference to the analysis by automated algorithms, unipolar limb leads obtained via the WCT with their smaller amplitude deflections would not present a problem. Also for visual assessment and manual measurements the current technology generated ECG tracings, with their higher signal/noise ratios, ensures a problem free environment (Figure 2 and 3). Moreover manual measurements (thought by some to be the gold standard) on enlarged ECG tracings can be done on a computer screen or electronic reading tablet (in cases of scanned ECG hardcopies), with reader-operated electronic cursors. Finally the possible concern that the familiarity of physicians with aVR/aVL/aVF will not be maintained with the substitution of these leads by VR/VL/VF recorded via the WCT is unfounded, since the latter have similar morphologies to the former [1]. (Figure 2 and 3).

But what would be the advantages of a substitution of the GCT with the WCT for recording the 3 unipolar limb leads? This would result in:

1. Uniformity in the recording of unipolar limb and precordial leads.
2. A sole, real and stable reference point for all unipolar ECG leads, which is theoretically, technologically, and physiologically appealing.
3. Comparability of the precordial leads and unipolar limb leads with the leads from total and limited body maps, esophageal leads, intracardiac leads, and any other conceivable leads, since all will employ the WCT [8,9].
4. Supplementation of 3 additional points of analysis to the above maps, and tying various ECG recording systems with the standard ECG.
5. Provision of a realistic assessment of the amplitude of Q-waves, ST-segment elevation and depression, or T-wave amplitude in conjunction with the precordial leads in myocardial infarction (MI) and ischemia. When e.g., an ECG showing a lateral MI is evaluated with ST-segment deviations involving the lateral precordial leads and aVL, one should constantly factor in that the amplitude of ST-segment deviation in aVL is augmented by 50%. Since the amplitude of ST-segment deviations is used in the standard ECG or limited or total body maps in estimating infarct size or area at risk, or in evaluation of reperfusion, the unreal amplitude of ST-segment deviation of aVL should be kept in mind. Similarly when in inferior, lateral, and apical or anteroapical combinations of MI are encountered, the lead aVF presents the same problems as lead aVL when is considered in concert with any of the precordial leads.
6. Realistic assessment of the value of the reciprocal ST-segment depression in lead aVR in the setting of an inferior and posterior MI; recent work has shown that such changes in aVR have diagnostic utility [12].
7. Realistic assessment of ST-segment elevation in lead aVR in patients with non-ST-elevation MI; recent work has detected important prognostic information in such changes in aVR [13].
8. Exploitation of the ST-segment elevation in lead aVR during exercise or pharmacologic stress testing; recent work has shown that such changes in aVR may detect significant stenosis of the left anterior descending coronary artery [14].
9. Realistic assessment of reciprocal ST segment elevation and ST segment depression in MI and myocardial ischemia.
10. Realistic assessment of all ECG components of the unipolar limb leads and their usefulness in diagnosis and therapy in future ECG systems. The underlying notion of the above arguments is that when employing quantitative ECG, one theoretically should not use in the calculation of sums measurements from leads that are not augmented (i.e., V1-V6) with leads that are augmented (i.e., aVR, aVL, and aVF).

It may be useful to rethink these issues, and impress on all interested in materia electrocardiographica that respect for the historical record, the power of the clinical convention, and the enormity of clinical experience employing aVR/aVL/aVF notwithstanding, there may be some merit in switching to VR/VL/VF. However, even if the recommendations of this Viewpoint are not adopted, merely delving in these issues may possibly generate innovations in the discipline. Perhaps a starting point can be that when summation of ECG potentials is used in practice or research, the values from aVR/aVL/aVF leads should be multiplied by 2/3 before proceeding with summation with the values from V1-V6 leads, since the limb and precordial leads have not been recorded against the same reference point. In this context values from VR/VL/VF can be incorporated as constituents of multi-lead mapping systems, since they were recorded via the same WCT. More is sure to come from contemplating the above proposal, while continuing to use the standard ECG.

## Figures and Tables

**Figure 1:**
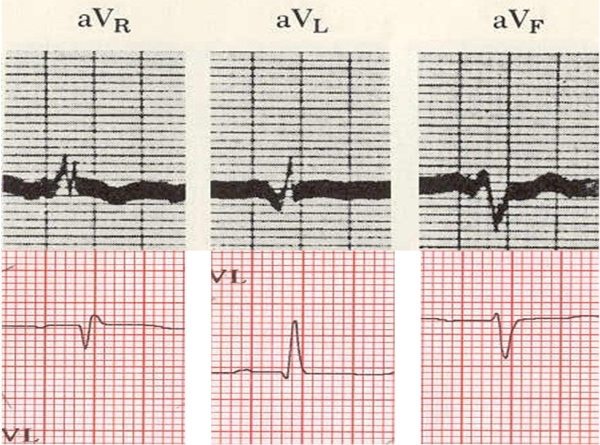
***Upper panel:*** Leads aVR, aVL, and aVF reproduced with permission from Littmann D. Textbook of electrocardiography, Harper & Row, New York, 1972, currently copyright of Lippincott Williams & Wilkins. ***Lower panel:*** Leads aVR, aVL, and aVF of the same patient as in Figure 2. Magnification of both ECGs is the same.

**Figure 2:**
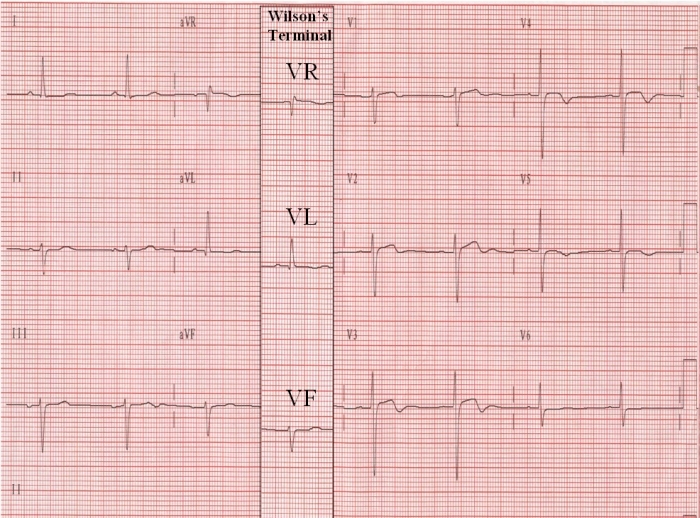
The column with leads VR, VL, and VF, recorded via the Wilson's terminal in the 2nd ECG, has been superimposed on the 1st ECG to aid in the comparison of leads VR, VL, and VF with aVR, aVL, and aVF.

**Figure 3:**
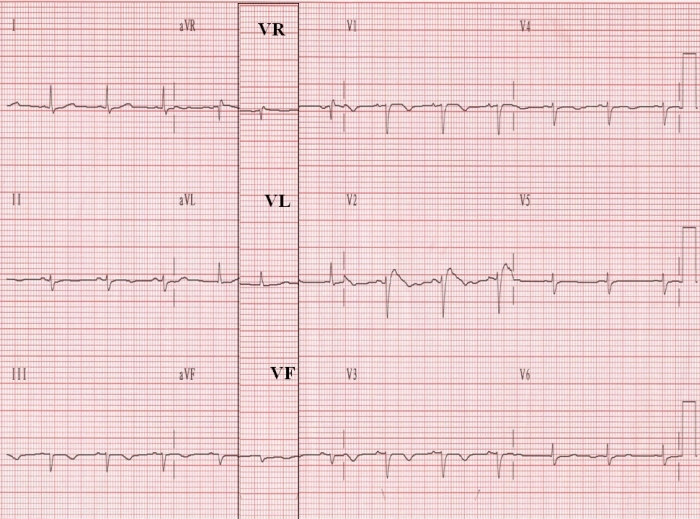
This "low voltage" ECG was recorded in an 85-year old woman with marked peripheral edema. The column with leads VR, VL, and VF, recorded via the Wilson's terminal in the 2nd ECG, has been superimposed on the 1st ECG to aid in the comparison of leads VR, VL, and VF with aVR, aVL, and aVF.
